# E-cadherin and K-ras: implications of a newly developed model of gastric cancer

**DOI:** 10.18632/oncoscience.379

**Published:** 2017-11-28

**Authors:** Jacob E. Till, Sam S. Yoon, Sandra Ryeom

**Affiliations:** Department of Cancer Biology, Perelman School of Medicine at the University of Pennsylvania, Philadelphia, PA, USA

**Keywords:** gastric cancer, KRAS, p53, E-cadherin, β-catenin

Mouse models have revolutionized our understanding of cancer and are key to preclinical development of cancer therapeutics. Despite the use of mouse models of cancer that closely recapitulate human disease, the FDA approves only 7.5% of new oncology drugs entering phase I clinical trials. One reason for these failures is that many preclinical studies target primary tumors while clinical trials typically involve patients with advanced or metastatic disease, which accounts for >90% of cancer-related mortality. This indicates the need for genetically engineered mouse models (GEMMs) of metastatic disease that faithfully recapitulate tumor progression [1]. We recently described one such GEMM of intestinal and diffuse (or mixed) type gastric adenocarcinoma (GA) [2]. Beyond the implications as a relevant preclinical model for drug development, this is the first model of mixed-type GA and suggests that gastric parietal lineage cells can be a cell of origin for GAs. Further, this model of GA provides direct evidence that E-cadherin loss can upregulate β-catenin signaling and that a single copy of the E-cadherin gene can attenuate oncogenic KRAS activity.

Our model of mixed type GA, with both intestinal- and diffuse-type lesions, combines conditional oncogenic Kras activation with previously described [3] loss of E-cadherin and p53, specifically in cells of the gastric parietal cell lineage (Atp4b+) and traces these cells with a YFP reporter. This model of GA is 100% penetrant with a median survival of 2.5 months compared to almost a year in the Kras wild-type model. Further, all mice develop regional lymph node metastases and lung metastases, half develop paratracheal lymph node metastases, and 20% developed liver metastases. The lung metastasis phenotype can be recapitulated with GA cell lines derived from these mice in an experimental flank tumor model of spontaneous metastasis. We provided a proof of concept of the preclinical utility of our model by demonstrating that treatment of these mice with a MEK inhibitor (PD0325901) extended median survival by 20 days.

It has long been hypothesized that E-cadherin loss can up-regulate β-catenin signaling. However several studies have shown that E-cadherin loss alone may not be sufficient and suggest that inhibition of the canonical WNT pathway destruction complex is also necessary [4]. Our study demonstrates that loss of E-cadherin is sufficient to drive increased β-catenin/TCF/LEF signaling in our model; yet, the status of the destruction complex is unknown. Further, cross-activation of canonical WNT signaling by the receptor tyrosine kinase (RTK)-Ras pathway has been well established [5]. Taken together, these data suggest that our β-catenin signaling phenotype results from both the loss of E-cadherin (releasing β-catenin from sequestration) and expression of oncogenic Kras.

In addition to RTK-Ras regulation of canonical WNT signaling, Ras has also been shown to regulate expression of E-cadherin [6]. However, few studies demonstrate the opposite interactions. Our study provides *in vivo* evidence that E-cadherin regulates the activity of oncogenic Kras by demonstrating that E-cadherin is not only an “invasion suppressor gene” but a “gatekeeper” of primary tumorigenesis in our model of gastric carcinogenesis. We demonstrate that one copy of E-cadherin is sufficient to attenuate Kras signaling at the transcriptional level and decreases both Kras activation as well as downstream Erk phosphorylation. Despite the strong oncogenic stimulus of constitutively activated mutant Kras and Trp53 loss in our model, E-cadherin expression dramatically decreases primary tumorigenesis with tumor progression occurring only after E-cadherin expression is lost. Dow et al observed a similar phenomenon with the adenomatous polyposis coli (Apc) tumor suppressor in colorectal cancer [7]. They showed that Apc knock-down is essential for oncogenic Kras and Trp53 loss driven colon adenocarcinoma in Lgr5+ cells. Given that APC and, now, E-cadherin both regulate β-catenin signaling, these data suggest that canonical WNT signaling may be crucial for mutant Kras and Trp53 loss-driven oncogenesis in both the gastric parietal cell lineage and Lgr5+ colon stem cells.

Finally, the definitive cell of origin of GA is still not known. Recent data has implicated Mist1+ cells [8] whereas our data implicates Atp-4b+ cells as the cell of origin for both the intestinal- and diffuse-type lesions. Further studies are necessary to determine if an Atp-4b+; Mist1+ cells may in fact be the true cell of origin in GA. If, in fact, a single cell of origin exists for both intestinal- and diffuse-type gastric cancer, this would contrast other cancers where each subtype is typically derived from a different cell of origin. Perhaps the GA histologic subtypes are not distinct entities but represent a spectrum of disease, that would explain why the histologic subtypes do not overlap with molecular classifications defined by recent studies. Our recently generated GEMM is the first model of mixed type GA and offers a valuable tool to investigate the mechanisms that underlie intestinal-type and diffuse- type lesions in this disease.

**Figure 1:**
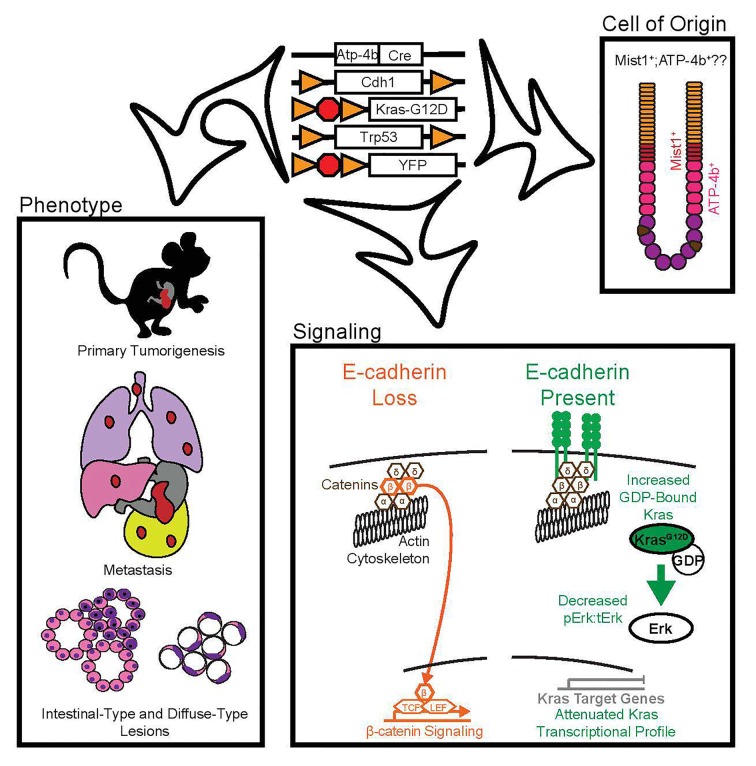
Schematic showing the genetics of our model of gastric cancer with the indicated phenotype, signaling pathways and possible cell of origin
